# Liposomal Systems as Nanocarriers for the Antiviral Agent Ivermectin

**DOI:** 10.1155/2016/8043983

**Published:** 2016-05-08

**Authors:** Romina Croci, Elisabetta Bottaro, Kitti Wing Ki Chan, Satoru Watanabe, Margherita Pezzullo, Eloise Mastrangelo, Claudio Nastruzzi

**Affiliations:** ^1^Department of Bioscience, University of Milan, 20133 Milan, Italy; ^2^Department of Life Sciences and Biotechnology, University of Ferrara, 44121 Ferrara, Italy; ^3^Program in Emerging Infectious Diseases, Duke-NUS Graduate Medical School, Singapore 169857; ^4^Institute of Biophysics, National Research Council, 20133 Milan, Italy

## Abstract

RNA virus infections can lead to the onset of severe diseases such as fever with haemorrhage, multiorgan failure, and mortality. The emergence and reemergence of RNA viruses continue to pose a significant public health threat worldwide with particular attention to the increasing incidence of flaviviruses, among others Dengue, West Nile Virus, and Yellow Fever viruses. Development of new and potent antivirals is thus urgently needed. Ivermectin, an already known antihelminthic drug, has shown potent effects* in vitro* on* Flavivirus* helicase, with EC_50_ values in the subnanomolar range for Yellow Fever and submicromolar EC_50_ for Dengue Fever, Japanese encephalitis, and tick-borne encephalitis viruses. However ivermectin is hampered in its application by pharmacokinetic problems (little solubility and high cytotoxicity). To overcome such problems we engineered different compositions of liposomes as ivermectin carriers characterizing and testing them on several cell lines for cytotoxicity. The engineered liposomes were less cytotoxic than ivermectin alone and they showed a significant increase of the antiviral activity in all the Dengue stains tested (1, 2, and S221). In the current study ivermectin is confirmed to be an effective potential antiviral and liposomes, as drug carriers, are shown to modulate the drug activity. All together the results represent a promising starting point for future improvement of ivermectin as antiviral and its delivery.

## 1. Introduction

Several emerging RNA viruses have been the cause of international epidemics within the past few years. The four Dengue virus (DENV) serotypes have considerably expanded their geographic distribution in recent years. With billions of people at risk, more than 50 million cases, and 12500–25000 deaths annually, DENV is considered an emerging pathogen in a growing number of countries [1]. In particular, the presence of four DENV (DENV 1, 2, 3, and 4) serotypes has complicated the design of vaccines because incomplete protection against one serotype may influence the disease outcome once infection is established by a different serotype, through a process referred to as antibody-mediated disease enhancement [2].

Since no specific antiviral treatment is available for flaviviruses, there is an urgent need for antiviral drugs.

Flaviviruses are enveloped viruses [3] with single-strand positive-sense RNA genome of about 11 kbs. There are more than 80 different flaviviruses [4] and most of them are important human pathogens, such as Yellow Fever virus (YFV), West Nile Virus (WNV) and its Australian variant Kunjin virus (KUNV), Japanese encephalitis virus (JEV), tick-borne encephalitis virus (TBEV), Saint Louis encephalitis virus (SLEV), and DENV. The C-terminal domain of nonstructural protein 3 (NS3) has an ATP-dependent helicase activity that maintains viral replication and for that reason is a good target for designing selective inhibitors of viral replication as therapeutic intervention [5].

Mastrangelo et al. [6] identified the widely used antihelminthic drug ivermectin as a molecule able to inhibit the NS3 helicase activity of several flaviviruses* in vitro* at submicromolar concentrations. Most importantly, ivermectin proved to be a selective inhibitor of the replication of YFV [6] and, although less efficiently, of other flaviviruses such as DENV, JEV, and TBEV. Ivermectin is now under clinical trials on humans, as a DENV therapeutic, at Mahidol University in Thailand [7].

Ivermectin is the common name of 22,23-dihydroavermectin B1, a semisynthetic derivative of avermectin family. It is a potent endo- and ectoparasitic agent with a broad spectrum of activity; its clinical use has been greatly recognized up the top award in science, being the Nobel Prize in Medicine and Physiology 2015 awarded to William C. Campbell and Satoshi Ōmura for their discovery of avermectin [8].

The pharmacokinetic behavior of avermectin depends upon the route of administration, the formulation used, the animal species, and pathophysiological status of the host. It is established that subcutaneous injection is the most efficient route for ivermectin administration in terms of drug bioavailability in sheep, cattle, and goats when compared with oral and topical administration [9].

Pharmaceutical technology has been applied to develop different drug formulations and delivery systems to optimize the pharmacological availability of ivermectin. The most promising approach for improving formulation lies in innovative delivery systems using carriers with defined physicochemical properties, such as liposomes, microemulsion, and polymeric micelles.

Although the efficacy of ivermectin has been established in humans against several parasite diseases, the antiviral properties of this compound are not yet exploited, mainly due to its complex chemical structure which cannot be easily chemically modified. The lack of appropriate formulations which could improve cellular internalization of ivermectin reduces the unfavorable effect of the drug [10].

According to these considerations, the current paper reports the development of engineered liposome formulations for possible clinical use of ivermectin. In particular, this study describes the production and the characterization of liposomes with ivermectin followed by subsequent tests of their antiviral activity and cytotoxicity in different cell lines.

Our results show that ivermectin, when delivered through liposomes, reduces the cytotoxicity and, at the same time, inhibits DENV replication with EC_50_ values in the micromolar range improving the performance of ivermectin alone.

## 2. Experimental Section

### 2.1. Chemicals

#### 2.1.1. Liposome Preparation

Highly pure phosphatidylcholine (PC) 90% from soybean (Phospholipon 90G Lipoid, Germany); cholesterol 97% (Fluka, Germany); dimethyldioactdecylammonium bromide (DDAB) (Sigma-Aldrich, UK); dimethyldioctadecylammonium chloride (DDAC) (Sigma-Aldrich, UK); and ivermectin (Sigma-Aldrich, UK) are used. The determination of entrapment efficiency was conducted with Sepharose 4B (Pharmacia, Uppsala, Sweden) and Isotonic Palitzsch Buffer (pH 7.44) as previously described [11]. For TLC analysis, iodine was used (Fluka, Germany).

#### 2.1.2. Cytotoxicity Test

2′-c-Methylcytidine (2-cmc) was from Sigma-Aldrich, UK. All the other reagents and solvents were from Sigma-Aldrich, UK, having analytical grade.

### 2.2. Liposome Preparation and Characterization

Liposomes were prepared by a minor modification of the ethanol injection procedure [11]. Typically, ivermectin was solubilized in ethanol (final concentration 50 mM) and different volumes were mixed with an ethanolic solution of PC 30–180 mM, plus cholesterol (10 mM) or DDAB (5–10 mM), in order to obtain the final required ivermectin concentrations. 500 *μ*L of the resulting ethanolic solution was injected by a syringe pump (500 *μ*L/min) into 4.5 mL of double-distilled water under magnetic stirring (300 rpm).

Liposome size analysis with photon correlation spectroscopy (PCS), the cryogenic transmission electron microscopy (cryo-TEM) analysis, and the determination of encapsulation efficiency were performed as previously described [11].

### 2.3. Cells and Viruses

All the cell lines were grown at 37°C in a humidified atmosphere of 5% CO_2_ with the addition in the medium of 100 U penicillin/mL and 100 *μ*g/mL streptomycin.

African Green Monkey Kidney Epithelial Cells 118 (Vero-118) were grown in IMDM (Life Technologies) supplemented with 10% or 1% fetal bovine serum (FBS). Mouse Leukaemic Monocyte Macrophage cells (RAW 264.7) were grown in DMEM (Life Technologies) supplemented with 10% or 2% FBS, 2 mM L-glutamine, 20 mM HEPES, 0.075 g/L sodium bicarbonate, and 1 mM sodium pyruvate. African Green Monkey Kidney Epithelial Cells F (Vero-F) and Baby Hamster Kidney (BHK) cells were cultured in DMEM (Life Technologies) containing 10% or 2% FBS. Hepatocellular carcinoma cells (Huh-7) were grown in DMEM (Life Technologies) containing 10% Fetal calf serum (FCS).

Dengue virus strains were obtained from the early Dengue infection and outcome (EDEN) study in Singapore [12] with the following GenBank accession numbers: DENV 1 (EU081230) and DENV 2 (EU081177) [13, 14]. The mouse adapted strain of DENV 2 S221 was kindly provided by Dr. Sujan Shresta (La Jolla Institute for Allergy and Immunology, CA). All virus strains were grown in C6/36 cells. Virus stocks were titered by plaque assay and stored at −80°C.

### 2.4. Cytotoxicity Determinations 

#### 2.4.1. Methylene Blue Assay

Cells were seeded (2 × 10^4^ cells/well) in 96-well plates either in the absence (controls) or in the presence of ivermectin (or 2-cmc taken as reference antiviral compound) at different concentration (0.2–25.0 *μ*M). After 4 days of cell culture at 37°C (35°C for Vero-118), the medium of each well was removed. 200 *μ*L of ethanol 70% was added to all wells and after overnight incubation it was removed. 50 *μ*L of filtered 1% (w/v) methylene blue in PBS was added to each well. After 1 h excess dye was removed. The cell layer, still stained with methylene blue, was examined microscopically. The wells were analysed giving a score to each well (high value: white; low value: blue).

#### 2.4.2. MTS Assay

Cytotoxicity was determined using the MTS [3-(4,5-dimethylthiazol-2-yl)-5-(3-carboxymethoxyphenyl)-2-(4-sulfophenyl)-2H-tetrazolium] assay. To this aim, the cells were seeded (5 × 10^4^ cells/well) in 96-well plates either in the absence (controls) or in the presence of ivermectin at different concentration (0.2–25.0 *μ*M). After 4 days of cell culture at 37°C a MTS/phenazine methosulphate (PMS) stock solution (2 mg/mL MTS (Promega) and 46 g/mL PMS (Sigma-Aldrich) in PBS at pH 6.0–6.5) was diluted 1/20 in MEM (Life Technologies). To each well, 75 *μ*L of MTS/PMS solution was added and the optical density (OD) was read at 498 nm 2 h later. The percentage of cell viability was calculated as (OD_treated_/OD_CC_) *∗* 100, where OD_CC_ is the OD of uninfected untreated cells and OD_treated_ are uninfected cells treated with compound.

#### 2.4.3. CC_50_ Calculation

The half maximal cytotoxicity concentration (CC_50_) was defined as the compound concentration that reduces the number of viable cells by 50% and it was calculated based on the nonlinear regression dose response sigmoidal curve drawn in GraphPad Prism.

### 2.5. Antiviral Assay in Human Huh-7 Cells Infected with DENV Viruses

10^5^ cells were infected with DENV 1, DENV 2, or DENV 2 mouse adapted S221 strain at MOI 0.3, in the presence of either ivermectin alone or ivermectin contained in liposomal formulations for 1 h. Inocula were discarded and replaced with maintenance media containing the same compounds and kept for 48 h. After 2 days, cell morphology was observed under microscope before collecting supernatant that was used for plaque quantitation assay. The virus titer without drug treatment routinely reached more than 10^5^ pfu/mL, representing the standard infection level in Huh-7 cells [15].

#### 2.5.1. EC_50_ Calculation

Half maximal effective concentration (EC_50_) was defined as the compound concentration that reduces the virus effect by 50% and it was calculated based on the nonlinear regression dose response sigmoidal curve drawn in GraphPad Prism.

## 3. Results and Discussion

### 3.1. Liposomes Preparation and Characterization

The preparation of liposomes was accomplished by ethanol injection since this method allows single-step formation of liposomes with a narrow size distribution, without degradation or oxidation of lipids or drugs. We used the “controlled” injection method in which the ethanolic solution of lipids is injected into the water phase via a syringe pump at a controlled rate, allowing precise ethanol diffusion in water. The mixing of ethanol with water is indeed the crucial step leading to lipid molecule self-assembly, driving liposome formation [11].

**Figure 1:**
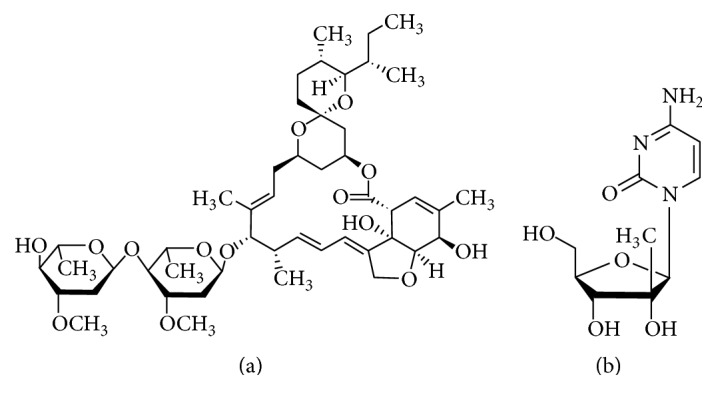
Chemical structure of ivermectin (a) and 2′-c-methylcytidine (b).

In order to optimize the formulation of liposomes for ivermectin (Figure 1(a)), we explored the roles of different experimental parameters on liposome characteristics, such as (a) lipid composition, (b) type of cationic surfactant, (c) PC content, and (d) ivermectin content.

Table 1 reports the complete composition of produced liposomes together with a unique identification code for each formulation.

**Figure 2:**
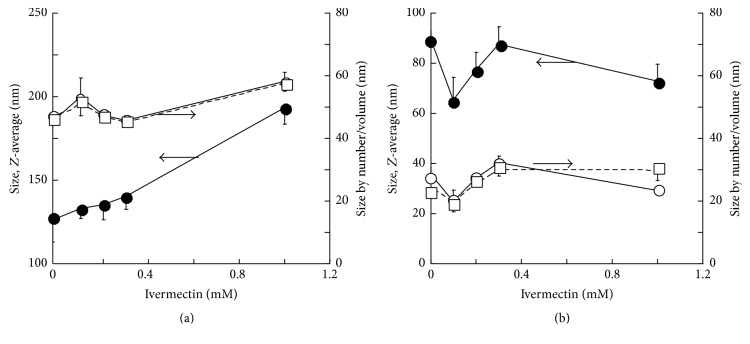
Effect of ivermectin content on the dimensional characteristics of different liposomal formulations (#PC9-Ch1-ive0.1, #PC9-Ch1-ive0.25, #PC9-Ch1-ive0.5, and #PC9-Ch1-ive1.0 in (a); #PC9-Br1-ive0.1, #PC9-Br1-ive0.25, #PC9-Br1-ive0.5, and #PC9-Br1-ive1.0 in (b)). Empty liposomes composed of PC/cholesterol (a) and PC/DDAB (b). Data refers to *Z*-average (filled circles, left *y*-axis), mean by number (open squares, right *y*-axis), and mean by volume (open circles, right *y*-axis). The arrows indicate the *y*-axis relative to the arrowed lines.

The effect of lipid composition and drug concentration was also investigated.  Figure 2 summarizes the results of photon correlation spectroscopy (PCS) measurements of liposomes composed of neutral lipids (PC/cholesterol (a)) and cationic lipids (PC/DDAB (b)). The effect of different amount of ivermectin (from 0.1 to 1.0 mM) on the dimension of liposomes is also shown in Figure 2.

The obtained results clearly indicate that cationic liposomes are smaller than respective neutral formulations; for instance, cationic liposomes containing 1.0 mM ivermectin have a *Z*-average of 72 nm, while for the neutral ones *Z*-average is 193 nm. This effect is due to molecular or physical interactions in conjunction with an increase in membrane fluidity induced by DDAB. In fact cationic lipids could facilitate electrostatic interactions of charged head groups. These interactions could promote size reduction by decreasing the radius of curvature of lipid bilayer as a result of phospholipid-cationic head group contact.

**Figure 3:**
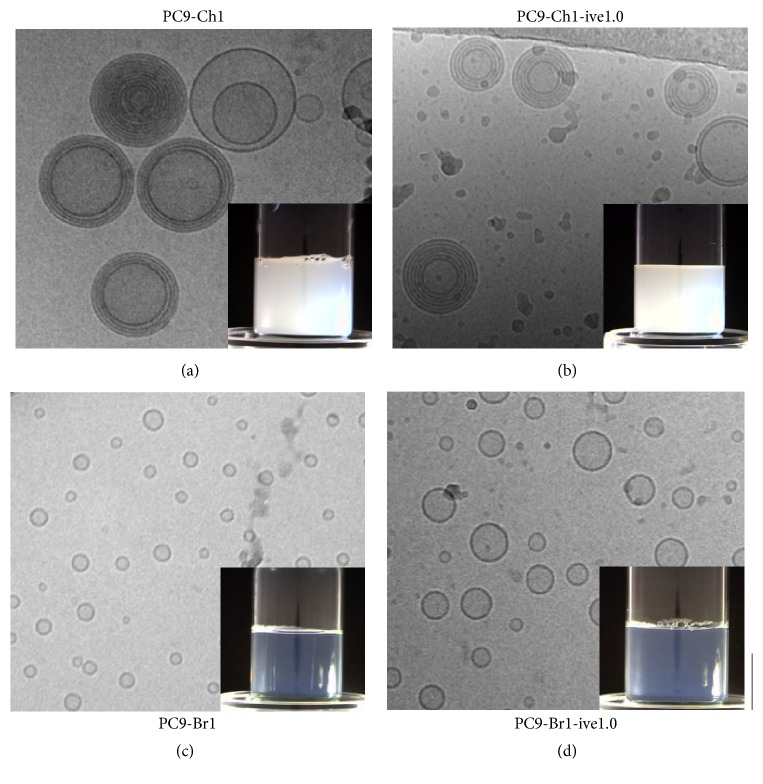
Cryo-TEM analysis of liposomal formulations, namely, #PC9-Ch1 (a), #PC9-Ch1-ive1.0 (b), #PC9-Br1 (c), and #PC9-Br1-ive1.0 (d). Bar corresponds to 700 nm for (a) and (b) and 100 nm for (c) and (d). For identification and chemical composition of liposomal formulation, see Table 1. In the insets the macroscopic appearance of the formulation is also reported.

Since the performance of liposomes, such as drug release or cell uptake, is influenced by morphological characteristics, an accurate microscopic characterization is mandatory before any biological evaluation. Results of PCS measurements were compared to those provided by cryo-TEM analysis. This technique enables the imaging of liposome architecture to evaluate its morphology together with an estimate of liposome size. The cryo-TEM images reported in Figure 3 demonstrate that both empty ((a), (c)) and ivermectin-loaded liposomes ((b), (d)), obtained by the “controlled” ethanol injection, had a spherical shape. The presence of a limited number of oligolamellar vesicles (typically 2–4 lamellae) in neutral liposome samples is evident ((a) and (b)). Microscopic images confirm that cationic liposomes are generally smaller than neutral ones and almost entirely composed of unilamellar vesicles with a size smaller than 100 nm in diameter (in agreement with PCS analysis).

**Figure 4:**
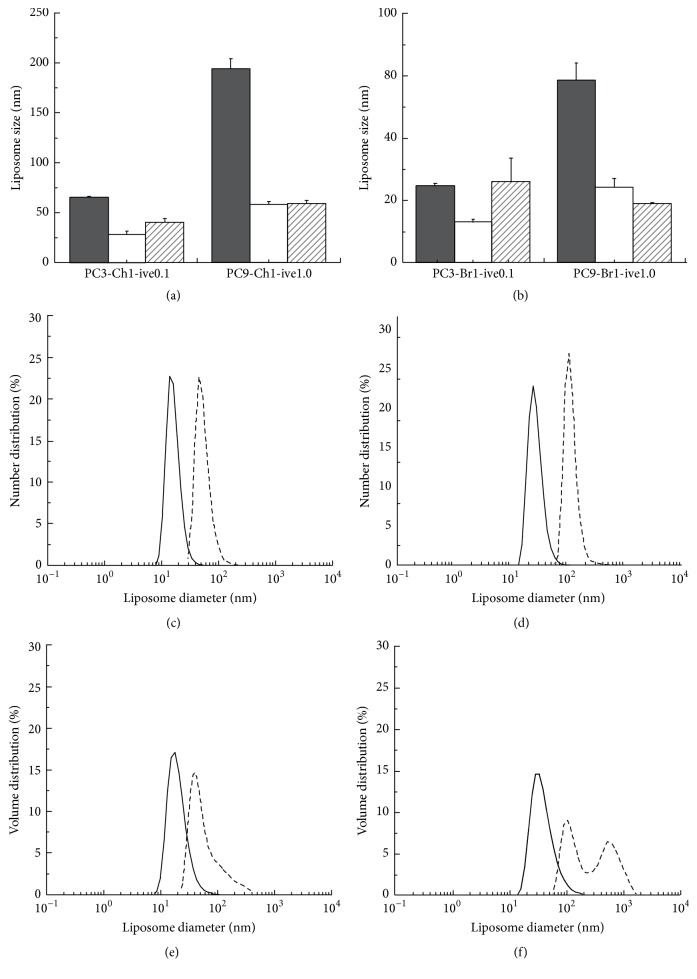
Effect of PC content and liposomal composition on mean diameters ((a), (b)) and size distribution ((c)–(f)) of the indicated ivermectin formulations. Bars in (a) and (b) correspond to *Z*-average (filled bars), mean by number (open bars), and mean by volume (striped bars). Curves in (c) and (e) correspond to #PC3-Ch1-ive0.1 (plain lines) and #PC9-Ch1-ive1.0 (dashed lines) liposomes; curves in (d) and (f) correspond to #PC3-Br1-ive0.1 (plain lines) and #PC9-Br1-ive1.0 (dashed lines) liposomes.

As further characterization, the effect of PC content on liposome dimension in the produced ivermectin-loaded liposomes was considered.  Figure 4 shows that an increase of PC from 3 mM to 9 mM causes the growth of the *Z*-average of neutral liposomes from 65 nm to 193 nm and, in case of cationic liposomes, from 30 nm to 72 nm.

**Figure 5:**
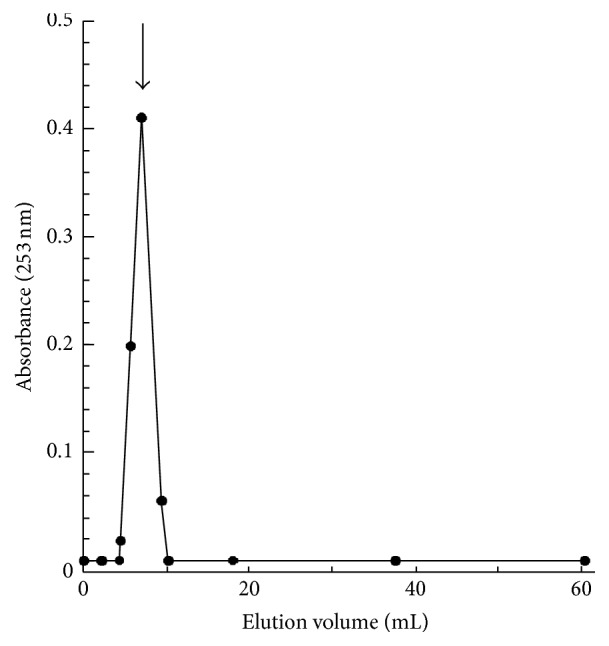
Elution profile of #PC9-Ch1-ive1.0 liposomes on Sepharose 4B gel-filtration column (length: 10 cm, diameter: 1 cm, and flow rate: 160 *μ*L/min). The amount of ivermectin associated with liposomes was determined from the optical density at 253 nm. The solid arrows indicate void volume fractions, including liposome-entrapped ivermectin.

The loading efficiency of liposomes for ivermectin was determined by size exclusion chromatography. As an example, the elution profile of ivermectin-loaded liposomes (#PC9-Ch1-ive1.0), obtained after Sepharose 4B chromatography, is shown in Figure 5. The elution profile shows a single well-resolved peak. The peak (indicated by the solid arrow) contains ivermectin-loaded liposomes; no peak was detected for free ivermectin, indicating that almost all ivermectin was associated with the liposomes. The presence of ivermectin in liposome peak was demonstrated by recording the UV spectrum of the corresponding fractions at 253 nm.

Therefore, the ivermectin loading yield was estimated by comparing the absorption at 253 nm of isolated liposomes with ivermectin standard solutions. The yield of ivermectin loading for liposomes prepared with both 3 mM and 9 mM PC was almost higher than 98% (data not shown). Such result indicates that the high liposome loading efficacy is achieved when ivermectin liposomes were produced by “controlled” ethanol injection method.

### 3.2. Ivermectin and Liposomes Cytotoxicity

**Figure 6:**
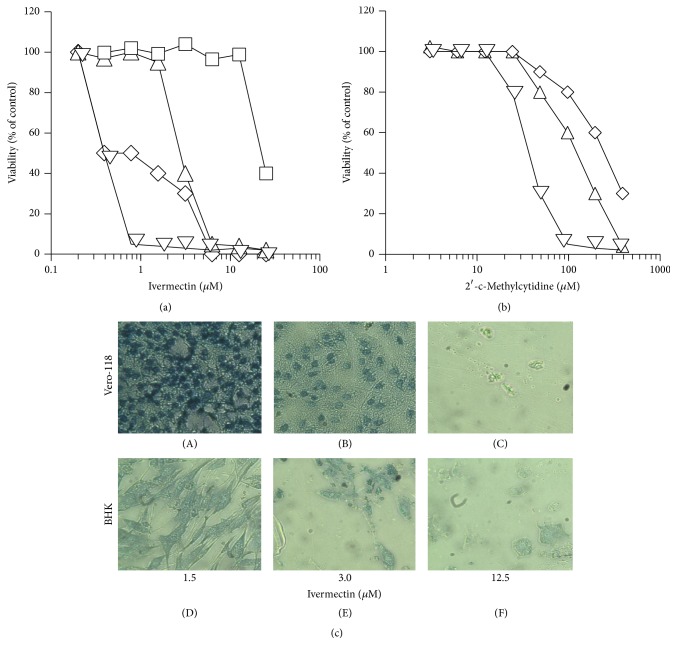
Effect of free ivermectin on the viability of BHK (diamonds), Vero-118 (upward triangles), Vero-F (squares), and RAW (downward triangles) cell lines (a). For comparison, in (b), the experiments are reported, conducted using 2′-c-methylcytidine, employed as reference antiviral compound, on BHK (diamonds), Vero-118 (upward triangles), and RAW (downward triangles) cell lines. (c) Optical microphotograph of Vero-118 ((A), (B), and (C)) and BHK ((D), (E), and (F)) cells treated with the indicated concentration of ivermectin. Data represent the average of 2 experiments.

To test the cytotoxicity of ivermectin, different cell lines were treated with several concentrations of the drug (0.2–25 *μ*M). Ivermectin on BHK and RAW cells shows CC_50_ < 1.5 *μ*M (BHK CC_50_ = 1.5 *μ*M, RAW CC_50_ = 0.8 *μ*M; Table 2) and on Vero-118, Vero-F, and Huh-7 has higher CC_50s_ (Vero-118 CC_50_ = 5.7 *μ*M, Vero-F CC_50_ = 23.6 *μ*M, and Huh-7 CC_50_ = 7.3 *μ*M, Table 2) (Figure 6(a)). In comparison to 2-cmc, already known viral polymerase inhibitor [16] (Figure 1(b)) ivermectin is 10 times more cytotoxic (Figure 6(b); Table 2).

**Figure 7:**
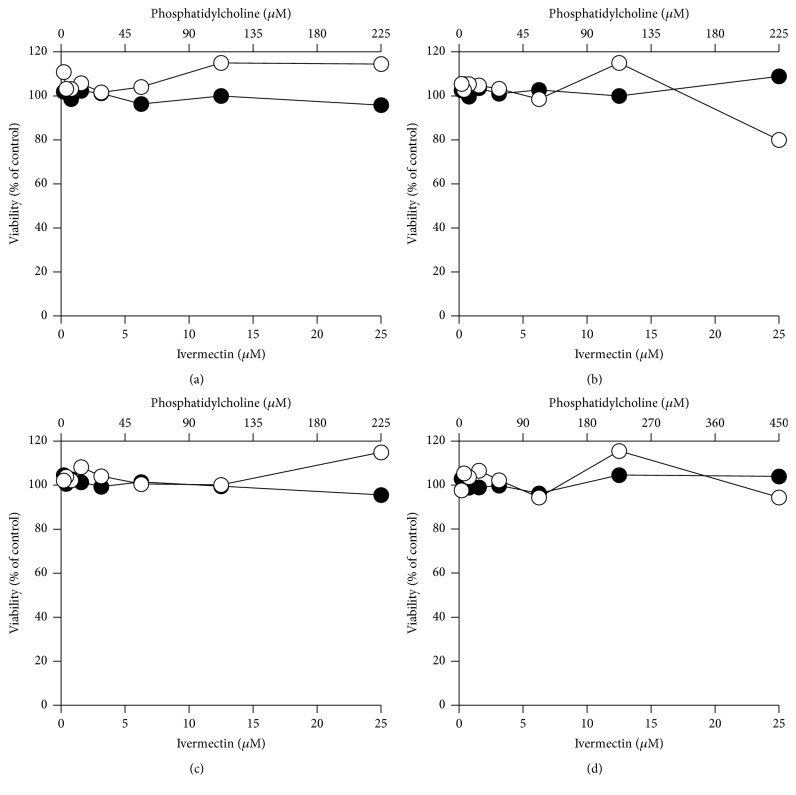
*In vitro* cytotoxicity effect of ivermectin formulated in liposomes (closed circles) on Vero-F cells. For comparison the same experiments were performed using empty liposomal formulations (open circles). The following liposomal formulations were tested: #PC9-Ch1 and #PC9-Ch1-ive1.0 (a), #PC9-Br1 and #PC9-Br1-ive1.0 (b), #PC9-Br0.5 and #PC9-Br0.5-ive1.0 (c), and #PC18-Br1 and #PC18-Br1-ive1.0 (d). The phosphatidylcholine concentration (top *x*-axis) is related to empty liposomes while the ivermectin concentration (bottom *x*-axis) is related to ivermectin liposomes. Details on the identification codes and liposomal compositions are included in Table 1. Data represent the average of 2 determinations.

**Figure 8:**
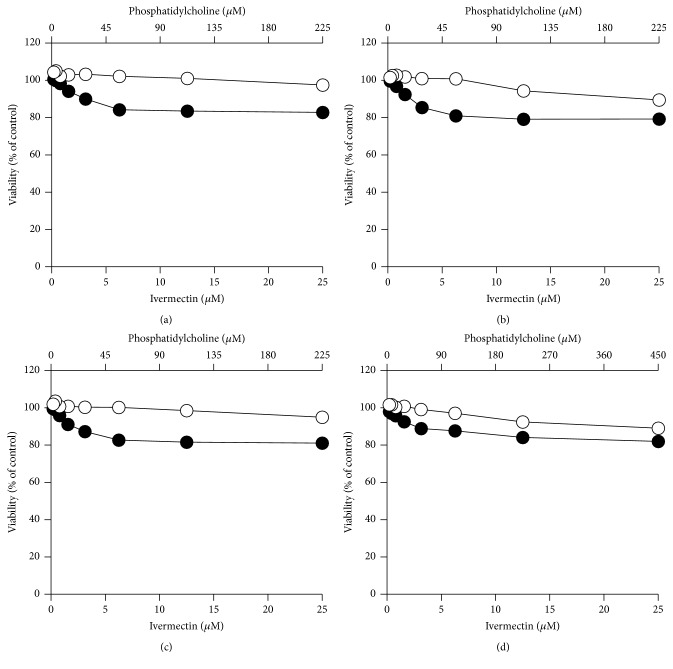
*In vitro* cytotoxicity effect of ivermectin formulated in liposomes (closed circles) on Vero-118 cells. For comparison the same experiments were performed using empty liposomal formulations (open circles). The following liposomal formulations were tested: #PC9-Ch1 and #PC9-Ch1-ive1.0 (a), #PC9-Br1 and #PC9-Br1-ive1.0 (b), #PC9-Br0.5 and #PC9-Br0.5-ive1.0 (c), and #PC18-Br1 and #PC18-Br1-ive1.0 (d). The phosphatidylcholine concentration (top *x*-axis) is related to empty liposomes while the ivermectin concentration (bottom *x*-axis) is related to ivermectin liposomes. Details on the identification codes and liposomal compositions are included in Table 1. Data represent the average of 2 determinations.

**Figure 9:**
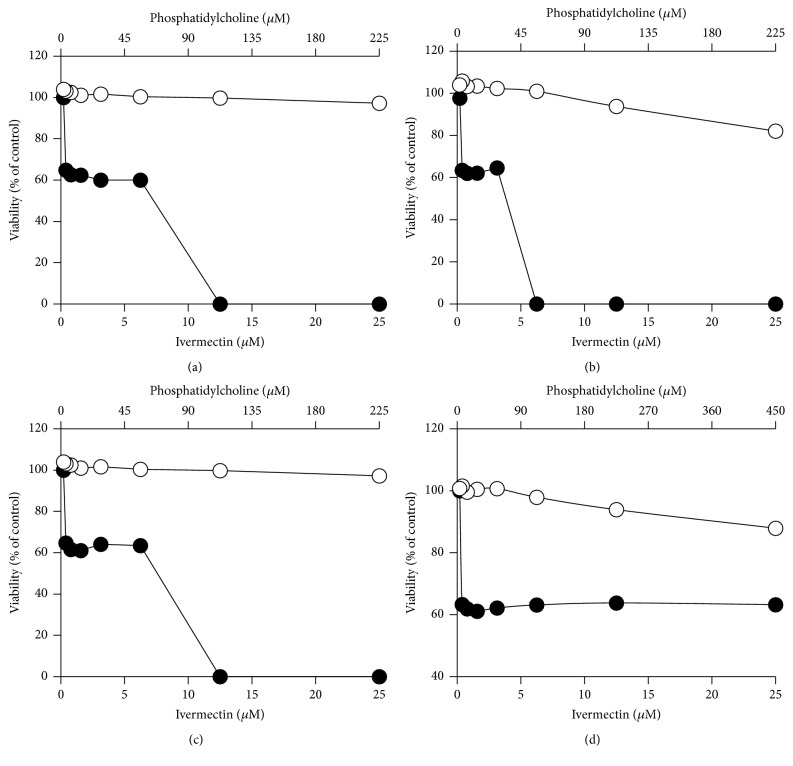
*In vitro* cytotoxicity effect of ivermectin formulated in liposomes (closed circles) on BHK cells. For comparison the same experiments were performed using empty liposomal formulations (open circles). The following liposomal formulations were tested: #PC9-Ch1 and #PC9-Ch1-ive1.0 (a), #PC9-Br1 and #PC9-Br1-ive1.0 (b), #PC9-Br0.5 and #PC9-Br0.5-ive1.0 (c), and #PC18-Br1 and #PC18-Br1-ive1.0 (d). The phosphatidylcholine concentration (top *x*-axis) is related to empty liposomes while the ivermectin concentration (bottom *x*-axis) is related to ivermectin liposomes. Details on the identification codes and liposomal compositions are included in Table 1. Data represent the average of 2 determinations.

**Figure 10:**
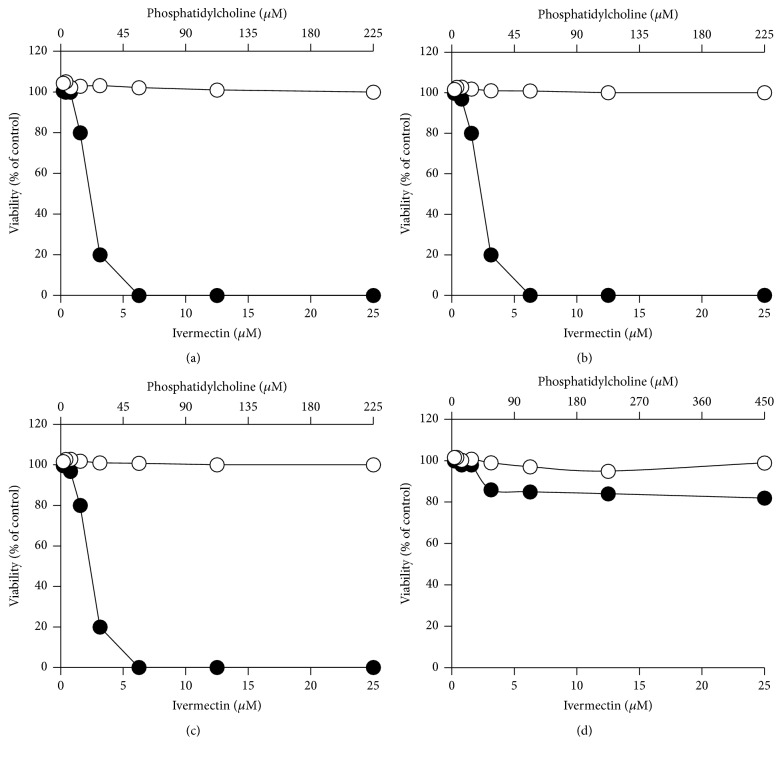
*In vitro* cytotoxicity effect of ivermectin formulated in liposomes (closed circles) on RAW cells. For comparison the same experiments were performed using empty liposomal formulations (open circles). The following liposomal formulations were tested: #PC9-Ch1 and #PC9-Ch1-ive1.0 (a), #PC9-Br1 and #PC9-Br1-ive1.0 (b), #PC9-Br0.5 and #PC9-Br0.5-ive1.0 (c), and #PC18-Br1 and #PC18-Br1-ive1.0 (d). The phosphatidylcholine concentration (top *x*-axis) is related to empty liposomes while the ivermectin concentration (bottom *x*-axis) is related to ivermectin liposomes. Details on the identification codes and liposomal compositions are included in Table 1. Data represent the average of 2 determinations.

In order to verify the cytotoxicity of ivermectin carried by liposomes, we tested #PC9-Ch1-ive1.0, #PC9-Br1-ive1.0, #PC9-Br0.5-ive1.0, #PC18-Br1-ive1.0, and the corresponding empty liposomes (#PC9-Ch1, #PC9-Br1, #PC9-Br0.5, and #PC18-Br1; Table 1) on Vero-F (Figure 7), Vero-118 (Figure 8), BHK (Figure 9), and RAW (Figure 10) cell lines. Ivermectin in liposomes shows reduced cytotoxicity in comparison to free ivermectin in all the cell lines. The highest effect is shown in Vero-118 with CC_50_ reduction of more than 5 times (from 5.7 *μ*M to more than 25.0 *μ*M). In BHK and RAW cells #PC9-Ch1, #PC9-Br1, and #PC9-Br0.5 reduced the ivermectin cytotoxicity 3 times (from 1.5 *μ*M to 4.3 *μ*M and from 0.8 *μ*M to 2.2 *μ*M, resp.) while #PC18-Br1-ive1.0 is completely noncytotoxic within the amount tested (Table 2). #PC3-Br1-ive0.1, #PC3-Ch1-ive0.1, #PC3-Cl1-ive0.1, #PC9-Ch1-ive1.0, #PC9-Br1-ive1.0, and the corresponding empty liposomes were tested on Huh-7. #PC3-Br1-ive0.1 is not cytotoxic in the concentration range tested (from 0.12 *μ*M to 10.0 *μ*M) whereas cytotoxicity around 10.0 *μ*M was observed for the others after 48 h of incubation.

In conclusion all the engineered liposomes are less toxic than ivermectin alone while the empty liposomes have no effect on cells cytotoxicity.

The reduced cytotoxicity is the result of several possible factors not completely clear yet. The most important is the use of aqueous solvent to solubilise the liposomes instead of the organic solvent that is necessary for ivermectin solubilisation.

### 3.3. Ivermectin and Liposomes Antiviral Activity on DENV

The antiviral activity of ivermectin, empty liposomes, and ivermectin liposomes was assayed* in vitro* on different virus strains, namely, DENV 1 (EU081230), DENV 2 (EU081177), and the mouse-adapted strain DENV 2 S221 [13, 14]. Since Dengue virus causes systemic infection and in particular the liver is implicated in humans, for the* in vitro* test, the Huh-7 cells were employed, representing one of the standard cell lines that are routinely used for the cell-based assay for Dengue infection.

Bassissi et al. [17] demonstrated that slight differences in formulation could change the plasma kinetics and efficacy of ivermectin in rabbits, subcutaneously given 0.3 mg/kg of ivomec (a commercial formulation of ivermectin) or ivermectin in liposomes. In order to test this finding, the* in vitro* antiviral activity of free ivermectin was compared to that of different liposomal formulations containing the drug. The antiviral assay was determined by adding the virus and ivermectin or ivermectin in liposomes at the same time. Particularly, it is important to underline that the routine setup commonly used for antiviral assays is indeed cotreatment (i.e., adding the compound to be tested concomitantly with the virus); thereafter drug/virus inoculum was removed and a further addition of the compound was made for 48 h incubation.

By this experimental approach, information about the antiviral activity of the compound (both direct and indirect acting) would be obtained. A different order of addition would provide preliminary insights into whether the compound is acting as a viral entry inhibitor (pretreatment) or virus replication inhibitor (posttreatment). Since ivermectin has been previously shown to act on viral helicase NS3 [6] and/or NS5-importin *α*/*β* [18], it would be ideal to test the different formulations of ivermectin by cotreatment assay as described in this paper.

**Figure 11:**
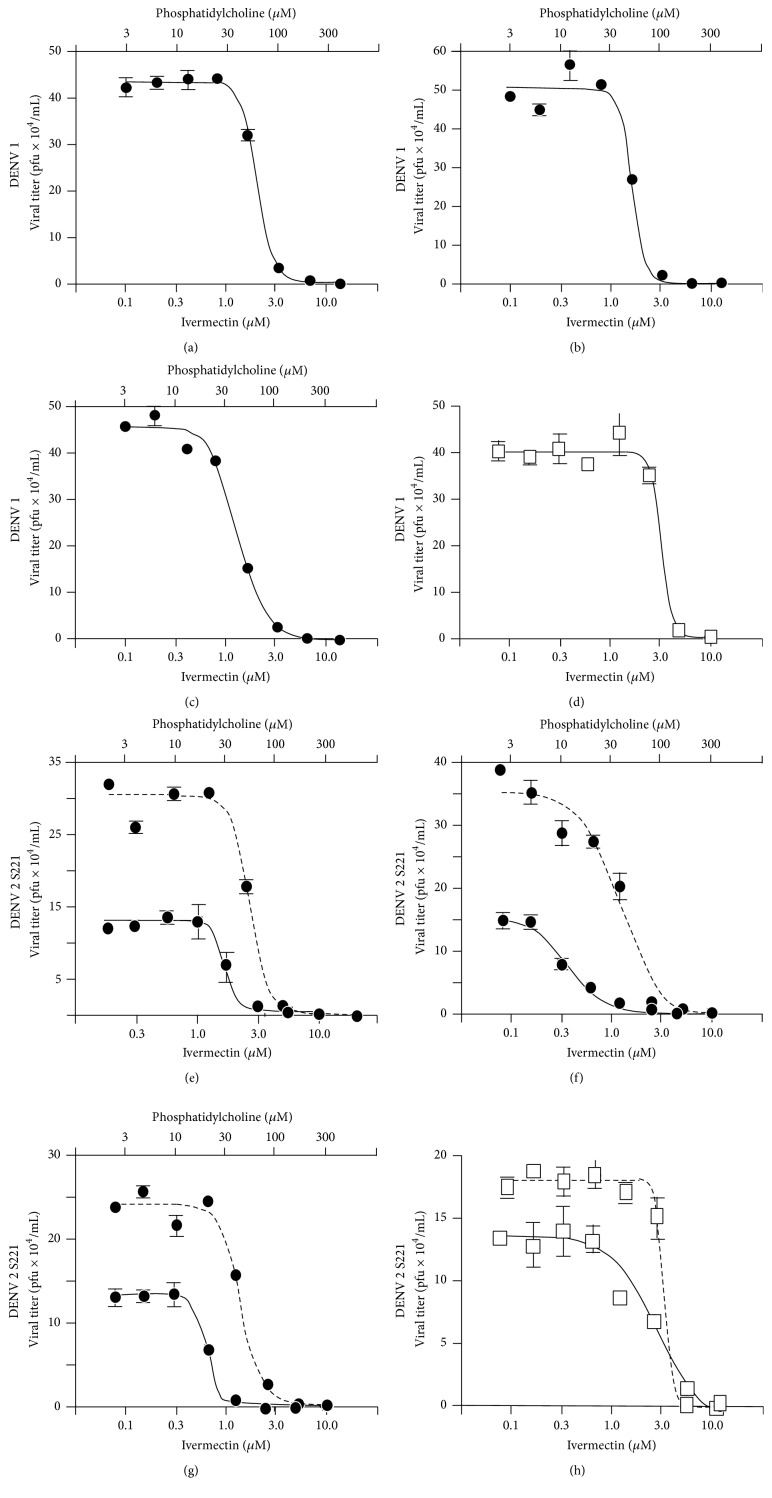
*In vitro* antiviral effect of ivermectin formulated in liposomes on Huh-7 cells infected with DENV 1. Ivermectin (d) and the following liposomal formulations were tested: #PC3-Ch1-ive0.1 (a), #PC3-Cl1-ive0.1 (b), and #PC3-Br1-ive0.1 (c).* In vitro* antiviral effect of ivermectin formulated in liposomes on Huh-7 cells infected with DENV 2 (plain lines) and DENV 2 mouse adapted S221 strain (dashed lines). The following liposomal formulations were tested: #PC3-Ch1-ive0.1 (e), #PC3-Cl1-ive0.1 (f), and #PC3-Br1-ive0.1 (g). For comparison, data relative to the free ivermectin are reported in (h). The related empty liposomes do not have any effect on the cells. The phosphatidylcholine concentration (top *x*-axis) is related to empty liposomes while the ivermectin concentration (bottom *x*-axis) is related to ivermectin liposomes. Details on the identification codes and liposomal compositions are included in Table 1. Details on the EC_50_ values are in Table 3. Data represent the average of 2 determinations ± SD.

**Figure 12:**
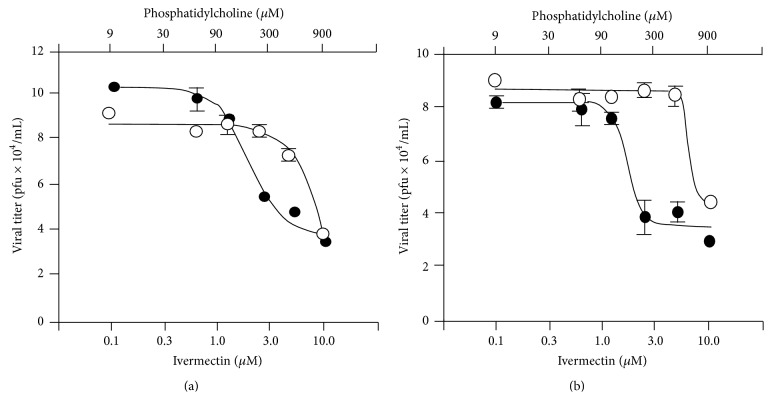
*In vitro* antiviral effect of ivermectin formulated in liposomes (closed circles) on Huh-7 cells infected with DENV 2. The following liposomal formulations were tested: #PC9-Ch1-ive1.0 (a) and #PC9-Br1-ive1.0 (b). For comparison, the infected cells were treated with the empty liposomal formulations (open circles). The phosphatidylcholine concentration (top *x*-axis) is related to empty liposomes while the ivermectin concentration (bottom *x*-axis) is related to ivermectin liposomes. Details on the identification codes and liposomal compositions are included in Table 1. Details on the EC_50_ values are in Table 3. Data represent the average of 2 determinations ± SD.

Notably the administration of ivermectin by a liposomal formulation yielded higher *C*_max_ and significantly faster absorption. This finding suggested that the use of liposomes could improve the* in vivo* efficacy of ivermectin [17]. Figures 11 and 12 report the activity of liposome preparations against DENV 1, 2, and S221 on Huh-7 cell line.

All the three liposome preparations tested against DENV 1 (#PC3-Ch1-ive0.1, #PC3-Cl1-ive0.1, and #PC3-Br1-ive0.1 (Figures 11(a), 11(b), and 11(c), resp.)) reduced EC_50_ in comparison to ivermectin alone (Figure 11(d)), with the best results observed for #PC3-Br1-ive0.1, leading to a considerable increase of the antiviral effect (from 3.7 *μ*M to 1.3 *μ*M; Figure 11(c)). The liposome preparations seem to be more effective against DENV 2. Results (plain line, Figures 11(e), 11(f), and 11(g)) suggest increasing efficacy of liposome packaged ivermectin. #PC3-Ch1-ive0.1 (Figure 11(g)) resulted in ~2 times reduction in EC_50_ (from 2.6 *μ*M to 1.3 *μ*M, Table 3) as compared to ivermectin alone (Figure 11(h)), while #PC3-Br1-ive0.1 and #PC3-Cl1-ive0.1 (Figures 11(e) and 11(f)) have shown great improvement in efficacy from 2.6 *μ*M to 0.3 *μ*M (5 times reduction in EC_50_). With low cytotoxicity (>10.0 *μ*M) and significant antiviral activity (0.3 *μ*M), #PC3-Br1-ive0.1 demonstrates being a good candidate for future* in vivo* experiments.  Figure 11 also shows the results for liposomes preparation against S221 mouse adapted strain (dashed lines). In this case, liposomes did not have significant improvement compared to free ivermectin. The respective empty liposomes have no effects on cell lines.

In order to analyse a higher concentration of ivermectin (1.0 mM), we tested liposomes #PC9-Ch1-ive1.0 and #PC9-Br1-ive1.0 and their respective empty liposomes (without ivermectin inside) (Figure 12) on Huh-7 infected with DENV 2. Empty liposomes do not have any effect on viral replication except at phosphatidylcholine concentrations >300 *μ*M. The dose response curve of infected cells treated with ivermectin liposomes is shifted to the left with respect to that of empty liposomes, confirming that ivermectin liposomes are able to reduce the plaque number, and so the active viruses. The liposomes containing 1 mM ivermectin slightly reduce EC_50_ when compared to free ivermectin (Table 3) but there is no significant difference in EC_50_ with respect to the previous tested liposomes (#PC3-Br1-ive0.1, #PC3-Cl1-ive0.1, and #PC3-Ch1-ive0.1) containing 1.0 mM ivermectin.

In conclusion we tentatively attribute the increase of antiviral activity (measured in terms of EC_50_ on DENV 2) from 2.6 *μ*M to 0.3 *μ*M to the fact that liposomes containing ivermectin could merge with cell membranes, facilitating the intracellular uptake of the drug. This hypothesis is supported by the fact that positively charged liposomes (i.e., those containing the cationic surfactants DDAC and DDAB) such as #PC9-Cl1-ive0.1 and #PC9-Br1-ive0.1 are the formulations displaying the highest antiviral activity (EC_50_ 0.3 and 0.6 *μ*M, resp.).

## 4. Conclusions

We demonstrate that ivermectin, when delivered through liposomes, reduced cytotoxicity up to 5 times. They can effectively inhibit DENV replication with EC_50_ values in the same range of ivermectin alone and even improve its activity in several formulations. The possibility of dissolving ivermectin into an aqueous solution thanks to the use of liposome as drug carriers is a new step towards the solution of its pharmacokinetics problems, in particular its high cytotoxicity. This could also amplify the spectrum of ivermectin activities.

The important improvement of ivermectin activity loading in liposomes with ions compositions creates a promising starting point for a future development of these nanocarriers which one day could be the possible solution to the lack of drugs against flaviviruses.

## Figures and Tables

**Table 1:** Liposomes identification code and composition.

Identification code	PC (mM)	Ch (mM)	DDAB (mM)	DDAC (mM)	Ivermectin (mM)
#PC3-Ch1-ive0.1	3.0	1.0	—	—	0.1
#PC3-Cl1-ive0.1	3.0	—	—	1.0	0.1
#PC3-Br1-ive0.1	3.0	—	1.0	—	0.1
#PC9-Ch1-ive0.1	9.0	1.0	—	—	0.1
#PC9-Ch1-ive0.25	9.0	1.0	—	—	0.25
#PC9-Ch1-ive0.5	9.0	1.0	—	—	0.5
#PC9-Ch1-ive1.0	9.0	1.0	—	—	1.0
#PC9-Br1-ive0.1	9.0	—	1.0	—	0.1
#PC9-Br1-ive0.25	9.0	—	1.0	—	0.25
#PC9-Br1-ive0.5	9.0	—	1.0	—	0.5
#PC9-Br1-ive1.0	9.0	—	1.0	—	1.0
#PC9-Br0.5-ive1.0	9.0	—	0.5	—	1.0
#PC18-Br1-ive1.0	18.0	—	1.0	—	1.0
#PC3-Br1	3.0	1.0	—	—	—
#PC9-Ch1	9.0	1.0	—	—	—
#PC9-Br1	9.0	—	1.0	—	—
#PC9-Br0.5	9.0	—	0.5	—	—
#PC18-Br1	18.0	—	1.0	—	—

PC: soya phosphatidylcholine; Ch: cholesterol; DDAB: Dimethyldioctadecylammonium bromide; DDAC: Dimethyldioctadecylammonium chloride.

**Table 2:** Half maximal cytotoxic concentration (CC_50_) expressed in *μ*M for ivermectin and ivermectin-loaded liposomal formulations.

Identification code	Vero-F	Vero-118	BHK	RAW	Huh-7
Ivermectin	23.6	5.7	1.5	0.8	10.0
2-cmc	n.d.	117.3	237.7	37.6	n.d.
#PC3-Ch1-ive0.1	n.d.	n.d.	n.d.	n.d.	~10.0^*∗*^
#PC3-Cl1-ive0.1	n.d.	n.d.	n.d.	n.d.	~10.0
#PC3-Br1-ive0.1	n.d.	n.d.	n.d.	n.d.	>10.0^*∗*^
#PC9-Ch1-ive1.0	>25.0	>25.0	8.4	2.2	~10.0^*∗*^
#PC9-Br0.5-ive1.0	>25.0	>25.0	8.5	2.2	n.d.
#PC9-Br1-ive1.0	>25.0	>25.0	4.3	2.2	~10.0^*∗*^
#PC18-Br1-ive1.0	>25.0	>25.0	>25.0	>25.0	n.d.

n.d. = not determined; ^*∗*^after 48 h of cell culture incubation.

**Table 3:** Half maximal effective concentration (EC_50_) expressed in *μ*M for ivermectin and ivermectin-loaded liposomal formulations.

Identification code	DENV 1	DENV 2	DENV 2 S221
Ivermectin	3.7	2.6	2.9
#PC3-Ch1-ive0.1	1.9	1.3	2.7
#PC3-Cl1-ive0.1	1.6	0.3	1.3
#PC3-Br1-ive0.1	1.3	0.6	1.4
#PC9-Ch1-ive1.0	n.d.	1.7	n.d.
#PC9-Br1-ive1.0	n.d.	1.7	n.d.

n.d.: not determined.

## Abbreviations

DENV 1–4: Dengue virus of types 1–4
YF: Yellow Fever
WNV: West Nile Virus
Cryo-TEM: Cryogenic transmission electron microscopy
PCS: Photon correlation spectroscopy
EC_50_: Half maximal effective concentration
CC_50_: Half maximal cytotoxicity concentration
TLC: Thin layer chromatography
FBS: Fetal bovine serum
FCS: Fetal calf serum
MOI: Multiplicity of infection
*C*_max_: Maximum serum concentration that a drug achieves in a specified compartment of the body after the drug has been administrated.
